# IL-1*β* and Allergy: Focusing on Its Role in Allergic Rhinitis

**DOI:** 10.1155/2023/1265449

**Published:** 2023-04-12

**Authors:** Han-Rui Wang, Shi-Zhuang Wei, Xiao-Yu Song, Yao Wang, Wen-Bin Zhang, Chao Ren, Ya-Kui Mou, Xi-Cheng Song

**Affiliations:** ^1^Department of Otorhinolaryngology, Head and Neck Surgery, Yantai Yuhuangding Hospital, Qingdao University, Yantai, China; ^2^Yantai Key Laboratory of Otorhinolaryngologic Diseases, Yantai Yuhuangding Hospital, Qingdao University, Yantai, China; ^3^Shandong Provincial Clinical Research Center for Otorhinolaryngologic Diseases, Yantai Yuhuangding Hospital, Yantai, China

## Abstract

Allergic rhinitis (AR) is a chronic upper airway immune-inflammation response mediated by immunoglobulin E (IgE) to allergens and can seriously affect the quality of life and work efficiency. Previous studies have shown that interleukin-1*β* (IL-1*β*) acts as a key cytokine to participate in and promote the occurrence and development of allergic diseases. It has been proposed that IL-1*β* may be a potential biomarker of AR. However, its definitive role and potential mechanism in AR have not been fully elucidated, and the clinical sample collection and detection methods were inconsistent among different studies, which have limited the use of IL-1*β* as a clinical diagnosis and treatment marker for AR. This article systematically summarizes the research advances in the roles of IL-1*β* in allergic diseases, focusing on the changes of IL-1*β* in AR and the possible interventions. In addition, based on the findings by our team, we provided new insights into the use of IL-1*β* in AR diagnosis and treatment, in an attempt to further promote the clinical application of IL-1*β* in AR and other allergic diseases.

## 1. Introduction

Allergic rhinitis (AR), one of the most common diseases in the world, is mediated by immunoglobulin E (IgE) produced by plasma cells after allergen exposure. It is a chronic upper airway inflammatory disease involving a variety of cells and cytokines [1]. AR is typically manifested as frequent attacks of nasal symptoms, including sneezing, runny nose, nasal itching, and nasal congestion, which seriously affect the quality of life [2]. Epidemiological surveys have shown that the incidence of AR is about 20-40%, even as high as 50% in some countries [3]. Currently, antihistamines, glucocorticoids, and immunotherapy are the mainstays of treatment for AR. Despite continuous improvement in therapeutic efficacy, the incidence of AR is still rising annually [4]. The pathogenesis of AR has not been well elucidated, and how to block the occurrence and development of AR more accurately remains a hot research topic.

Interleukin-1*β* (IL-1*β*), one of the core factors in the occurrence and development of inflammation, plays a key role in allergic diseases [5]. Studies have revealed that IL-1*β* may be closely related to allergen-induced inflammation and its clinical manifestations such as itching, redness, rashes, and runny nose. More studies have found that IL-1*β* level was significantly increased in allergic diseases [6], and blocking the IL-1*β* signaling pathway may reduce the severity of inflammation to a certain extent. Therefore, it has been proposed that IL-1*β* is expected to be a potential biomarker of AR. However, the poorly defined role and mechanism of IL-1*β* in AR and the inconsistencies in clinical sample collection and detection methods among different studies have limited the use of IL-1*β* as a clinical diagnosis and treatment marker for AR. Unfortunately, few studies have summarized recent research on the relationship between IL-1*β* and AR. Based on the findings of our own studies and in the literature, we elucidate the expression, cellular source, and action target of IL-1*β* in allergic diseases, focusing on recent advances in IL-1*β* in AR, in an attempt to further promote the clinical application of IL-1*β* in AR and other allergic diseases.

## 2. Effects of Allergen Exposure on IL-1*β*

The IL-1 cytokines mainly contain two members: IL-1*α* and IL-1*β*. These two proteins have the same gene loci and three-dimensional structures. As IL-1*α* is only locally expressed on the cell membrane of a limited number of cell types, it is difficult to detect in human blood and other body fluids, thus limiting its use in clinical practice [7, 8]. In contrast, IL-1*β* is more sensitive to immunity-related inflammation and can be secreted into body fluids (e.g., blood) with a detectable activity [9]. Accordingly, IL-1*β* has become the most studied member of the IL-1 family. Allergic reactions are triggered when skin or mucous membrane is exposed to allergens. Previous studies have shown that IL-1*β* is rapidly released from cells within 1–2 hours after allergen challenge [10, 11], peaks within 5–12 hours after challenge [12–14], and gradually returns to the basal level after 24 hours [15].

Nielsen et al. investigated the expression of IL-1*β* after exposure to allergens [16]. Contact hypersensitivity (CHS) models were established using female C57BL/6 (WT) mice, during which 0.15% dinitroflourobenzen (DNFB) was used as an allergen for three consecutive days. The generation of IL-1*β* in epidermis was detected at 6, 24 and 48 hours after challenge with the same dose of DNFB on day 24. It was found that the expression level of IL-1*β* increased rapidly after exposure to DNFB and peaked at 24 hours. Similarly, Enk et al. challenged BALB/c mice with DNFB as the allergen and also found that IL-1*β* showed a rapid increase at 1 h after challenge and continued to be highly expressed within 24 hours [17]. These *in vivo* findings in mice were consistent with the findings of Badorrek et al. and Boehm *et al.*, which showed that IL-1*β* was rapidly produced after exposure of human skin to allergens such as grass pollen or urushiol [14, 18]. Sim et al. also demonstrated that the body could release IL-1*β* after exposure to allergens, and IL-1*β* might be involved in the occurrence and development of inflammation [12].

According to Bachert C et al., AR is a persistent inflammation, and the expression of IL-1*β* varies in different stages of AR: the expression is significantly higher in the early and late stages, which may be related to the activation of T lymphocytes and endothelial cells and the further release of cytokines [19]. The production and activation pathways of IL-1*β* may be closely related to the NOD-like receptor thermal protein domain associated protein 3 (NLRP3) inflammasome [20]. Notably, in addition to the inflammatory response caused by the exposure of surface skin or mucous membrane to allergens, IL-1*β* also plays an important role in gastrointestinal allergic diseases induced by food allergens such as milk [21].

## 3. Changes of IL-1*β* in Allergic Diseases

Allergic disease is a specific inflammatory disease caused by the immune imbalance of the body. It is closely related to individual genetic and environmental factors. Such diseases include AR, allergic asthma, allergic conjunctivitis, and atopic dermatitis [22]. T helper cell type 1 (Th1)/T helper cell type (Th2) imbalance has been accepted as an important pathogenesis of allergic diseases [23]. Th2 cytokines (e.g., IL-4, IL-5, and IL-13) are significantly increased during the course of allergic diseases. IL-1*β*, as a key cytokine in the development of inflammation, has also been found to play an important role in allergic diseases such as AR, allergic asthma, and atopic dermatitis [18, 24–27]. However, it remains unclear whether IL-1*β* induces a Th1/Th2 imbalance or facilitates the reaction development. Although the ultimate effector site of the disease varies, the effects of IL-1*β* have been reported to be closely related to eosinophilia [28], IgE switching [29, 30], and Th2 inflammation [31, 32] in allergic diseases. In airway diseases in particular, the upregulation of IL-1*β* is positively correlated with airway remodeling caused by allergic inflammation [33]. When the IL-1*β* signaling pathway is specifically blocked, the activity of the smooth muscle cells involved in airway remodeling is significantly reduced, thereby inhibiting airway remodeling to some extent [34, 35]. In addition, IL-1*β* may also be involved in the onset of allergic conjunctivitis or food-induced gastrointestinal allergic diseases [36–38]. Most of the IL-1*β* showed a significant increase during the occurrence and development of these allergic diseases, which is consistent with the results of *in vitro* cell experiments and animal experiments, as shown in Table 1 [11–15, 17–19, 21, 24–27, 37–46]. Johnson et al. [46 ] and Groves et al. [47, 48] also used IL-1*β*-deficient mice to explore the mechanism of action via which the IL-1*β* signaling system is involved in allergic asthma and atopic dermatitis. Interventions (e.g., IL-1*β* receptor antagonists (IL-1Ra) and IL-1*β* inhibitors) on the IL-1*β* signaling system have also become research hotspots in the treatment of allergic diseases.

## 4. Relationships of IL-1*β* with Different Cells in Allergic Diseases

**Figure 1:**
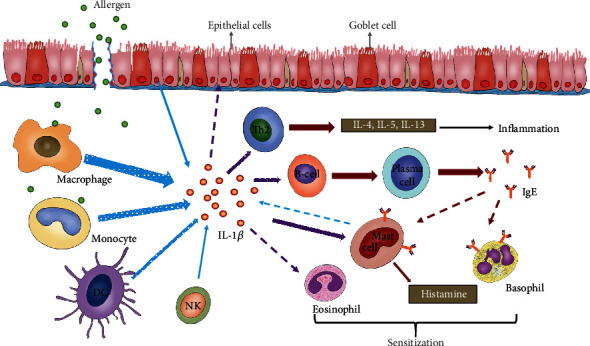
Possible cellular sources and effector cells of IL-1*β* following allergen challenge. The left side of the picture shows the possible source cells of IL-1*β*, and the thickness of the blue arrow suggests the primary and secondary relationship of the source; the purple arrow on the right side of the picture points to the possible effector cells of IL-1*β*; and the dashed arrows all suggest potential IL-1*β* related cells, but there is insufficient literature as a theoretical basis.

As shown in Figure 1, monocytes-macrophages are considered to be the main source of IL-1*β* in inflammatory diseases. In addition, almost all nucleated cells, such as B lymphocytes, natural killer (NK) cells, mast cells, and epithelial cells, can also produce IL-1*β* [49, 50]. It is reported that during inflammation, monocytes, macrophages, and lymphocytes present with increased IL-1*β* expression and secretion after the body is exposed to allergens [51–53]. In addition, some epithelial cells previously thought not to produce cytokines [54], such as epithelial cells in the respiratory tract, epidermis, and digestive tract, have also been reported to release IL-1*β* in allergic diseases [55, 56]. It was also found that nasal mucosal epithelial cells produced IL-1*β* after being stimulated by allergens [57], which was consistent with the findings in *in vitro* human nasal mucosa epithelial cell models exposed to allergen or in *in vivo* AR animal models [58, 59]. Therefore, how to precisely regulate IL-1*β* in the AR nasal mucosal epithelium has become a hot research topic.

Lymphocytes are often described as core cells in allergic diseases. As a lymphocyte stimulator, IL-1*β* can not only be secreted by B cells but also activate and strengthen the activity of B cells to a certain extent, thus promoting their differentiation into plasma cells and triggering the production of site-specific IgE [60, 61]. As a cytophilic antibody, IgE has high-affinity receptors on its surface, and when combined with effector cells such as mast cells and basophils, it can sensitize the organism. When these sensitized cells are exposed again to the corresponding allergen, they will trigger cell degranulation and the release of inflammatory mediators, leading to the development of allergic diseases such as allergic dermatitis, allergic asthma, and AR [62]. Therefore, IgE is often markedly increased in the serum or local secretions of patients with allergic reactions. Of course, IL-1*β* can also directly act on mast cells to enhance the release of cytokines and histamine secreted by mast cells and promote their response to other relevant cytokines, thereby assisting in the induction and maintenance of allergic reactions [63]. In addition, IL-1*β* also enhances the immune response mediated by Th2 cells, affecting the infiltration of Th2 cells in tissues and facilitating the high expression of cytokines such as IL-4, IL-5, and IL-13 [64]. Interestingly, it has also been reported that IL-1*β* seems to stimulate its own production and processing; when its precursor increases to a certain level, IL-1*β* is prone to induce an autoinflammatory state [65]. However, how does IL-1*β* induce and promote such diseases? Many studies have shown that the rationale of the above reactions is that the receptor for IL-1*β* (IL-1R) exists on the cell membrane of almost all cells [66]. Both *in vivo* and *in vitro* studies have shown that tissues or cells lacking or knocking down IL-1R will significantly reduce the inflammatory responses [67, 68]. However, the specific effects and mechanisms of IL-1*β* and its receptors on AR and other allergic diseases have not been fully elucidated and warrant further exploration.

## 5. IL-1*β* and AR

### 5.1. Cellular Research

Nasal epithelial cells (NEC) are among the first cells to be exposed to inhaled allergens and are also the main cells involved in AR. In many studies, IL-1*β* has been found to be significantly overexpressed in isolated human nasal epithelial cells (HNEpC) after allergen exposure [69–71]. Li et al. found that the significant increase in IL-1*β* in HNEpC after exposure to pollen might involve the integration of the ROS-NLRP3-Caspase-1-IL-1*β* signaling pathway [72]. However, such high expression can be inhibited by anti-inflammatory drugs such as corticosteroids and topical hormones [73, 74]. Min et al. also observed a significant increase in AR-related inflammatory markers when HNEpC was directly stimulated with IL-1*β* [75]. Therefore, all these *in vitro* studies demonstrated the important role of IL-1*β* in allergen-induced nasal mucosal inflammation.

### 5.2. Animal Research

In the *in vivo* animal models, Kim et al. used ovalbumin (OVA) to induce the AR models in 6-week-old BALB/c mice and found that the content of IL-1*β* in the serum and nasal mucosa after OVA challenge was significantly higher in the model group than in the control group, and the increase of IL-1*β* was closely related to eosinophils and mast cells in these tissues [76], which were consistent with the findings of another study [77]. In their subsequent experiment, Kim et al. further confirmed that caspase-1-dependent processing and maturation of IL-1*β* are the key processes leading to allergic symptoms in mouse models [78]. In addition, Wan et al. also used OVA plus alum to induce AR models in SD rats and found that IL-1*β* could promote the differentiation of Th17 cells and maintain the production of Th17-related cytokines, thereby enhancing the Th2 immune response, promoting B cells to produce IgE, and promoting eosinophils recruitment [79]. These *in vivo* studies have shown that IL-1*β* can affect the eosinophils and inflammatory/immune responses through different pathways and different mediators and is involved in the pathogenesis of AR.

There are currently numerous trials in animals that demonstrate the effectiveness of blocking IL-1*β* signaling in reducing allergic airway inflammation. Wei et al. used IL-1*β* inhibitors to intervene in OVA-induced allergic airway inflammation in guinea pigs and found that IL-1*β* inhibitors, to a certain extent, attenuated pathological allergic inflammation in the nasal mucosa and lung tissues of AR guinea pigs, similar to budesonide (a commonly used anti-inflammatory drug in clinical practice) [80]. In addition to direct inhibition of IL-1*β*, Willart et al. also found that IL-1R-deficient mice did not develop a Th2-dominant immune response after exposure to house dust mites, nor did they develop allergic airway inflammation [81]. In addition, the development of drugs such as the IL-1R antagonist anakinra (Kineret®), rionacept (Regeneron®; IL-1 trap), and canakinomab (Ilaris®; anti-IL-1*β* antibody) also suggests that blocking IL-1*β* signaling may be a potential complementary therapy for allergic airway inflammation [82, 83]. Of course, it is also been investigated in other allergic diseases. Nielsen et al. injected IL-1 receptor antagonist (IL-1Ra, (anakinra)) 12 hours before the challenge of mouse models and found that the allergic symptoms were significantly reduced after these mice were exposed to the potent contact allergen 2,4-dinitrofluorobenzene (DNFB) [16]. Keane-Myers et al. also treated mice with recombinant IL-1Ra (rIL-1Ra) to reduce the inflammatory response during allergen-induced conjunctivitis [84]. Therefore, when the IL-1*β* signaling pathway is specifically blocked, inflammation/immune response will be significantly reduced.

### 5.3. Clinical Research

The expression and possible mechanism of IL-1*β* in AR have been widely studied in *in vivo* mouse models and in *in vitro* cell models; however, only a small number of relevant clinical studies have been conducted. Compared with healthy volunteers without AR, AR patients had significantly higher expressions of IL-1*β* in nasal secretions, nasal mucosa, peripheral blood mononuclear cells, and serum [85–88]. However, Wagenmann et al. discovered that the expression of IL-1*β* was not stable and varied with the change of sampling time and method [11], which limits its clinical application. In addition, when the human body is exposed to allergens such as pollen, the increase of IL-1*β* can be accompanied by the increase of IL-1Ra [89]. However, Krouse et al. found that IL-1Ra had low expression in the early stages of allergen exposure [90]. These findings may be closely associated with the complex compensatory mechanisms of the antiallergic reaction of the human body. Taken together, the results of the human studies were consistent with those of *in vivo* animal models and *in vitro* cell models. While in clinical practice, the cut-off values defining IL-1*β* elevation were not clearly prescribed, we summarized the relevant information provided in the published articles, as shown in Table 2 [11, 13, 57, 85, 88, 90–94]. And we discovered that the current clinical applications on IL-1*β* are more focused on pediatric AR patients. Previous studies have indicated that AR in children is characterized by an increase in IL-1*β* and that excessive release of IL-1*β* can hasten the progression of the condition [26]. In a risk factor analysis of two cohorts of children with AR, Han et al. noted a 5.8-fold increased risk of developing moderate to severe AR when IL-1*β* >7.35 pg/ml in one study [88], and when IL-1*β* ≥7.98 pg/ml in the other research, the chance of developing moderate to severe AR rose by 4.7 times [91]. According to the aforementioned two research, IL-1*β* is a potential predictor of the possibilities for moderate to severe AR. In contrast, although IL-1*β* is also commonly elevated in adults with AR, its amounts are extremely variable, and there are no established cut-off values. Nevertheless, the present findings all imply that IL-1*β* can be utilised as a characteristic factor of AR inflammation to differentiate AR from controls and can also be applied to assess the efficacy of different medications on AR. Therapies that target IL-1*β* are also being implemented progressively. By building a biosensor, Lijuan et al. confirmed the feasibility of IL-1*β* as a potential therapeutic target for AR in 2021 [95]. The IL-1R antagonists anakinra (Kineret®), rionacept (Regeneron®; IL-1 trap), and canakinomab (Ilaris®; anti-IL-1*β* antibody), mentioned above, have not been studied in clinical trials alone in patients with AR, but they have been shown to be effective in treating rheumatoid arthritis, type 2 diabetes, and recurrent pericarditis [96–98]. Hence, the popularization and application of IL-1*β*-related targeted drugs to AR patients have a promising future. Before IL-1*β*-related drugs are widely available in the clinic, AR patients do not need to be overly anxious about when such treatments will be supplied; after all, AR is rarely life-threatening. At this stage, it has been demonstrated that a substantial majority of patients can effectively control their disease symptoms while using antihistamines or glucocorticoids. Without a sure, the advent of medications connected to IL-1*β* will provide us with more choices.

## 6. Conclusions and Prospects

IL-1*β* is involved in the occurrence and development of AR. It can be rapidly released from many cells immediately after allergen exposure to induce the production of histamine and itself. In addition, IL-1*β* can promote the adhesion and aggregation of a variety of leukocytes and then participate in the late-phase IgE-dependent inflammatory response [44]. Therefore, blocking the IL-1*β* action in hypersensitivity may reduce the severity of inflammation. Wei et al. demonstrated this therapeutic effect in a mouse model of allergic airway disease by administering an IL-1*β* inhibitor [80]. In addition to inhibiting the activity of IL-1*β*, IL-1 receptor antagonist (IL-1Ra) can also compete with IL-1*β* for binding to IL-1R, thereby blocking its downstream pathway [99]. IL-1 blockers, such as anakinra, have been used in clinical practice to intervene in various diseases, and many studies have confirmed their efficacy [100, 101]. However, such IL-1*β*-targeted drugs are rarely applied in the clinical treatment of AR, and most relevant studies were conducted in mouse models of allergic diseases, and no clinical study has been published so far.

**Figure 2:**
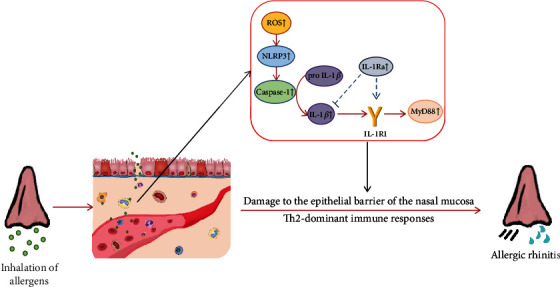
Possible pathogenesis of IL-1*β* in allergic rhinitis.

Similarly, the pathogenesis of AR has not been elucidated, and existing researches have considered that it mostly mediates disease development via the ROS-NLRP3-Caspase-1-IL-1*β* signaling pathway [72, 102]. In allergic airway illnesses, the mucosal epithelial barrier serves as the first defense line, and the collapse of the barrier is closely linked to this pathway [103]. Intervention at any target of this signaling was identified to reduce the expression of IL-1*β* and also to alleviate the damage of the epithelial barrier and improve the allergic symptoms and inflammatory response in AR patients [104–106]. Our team (https://csohns2022.sciconf.cn/cn/web/abstract-search/13734) used human-derived IL-1*β* to directly stimulate human nasal mucosal epithelial cells and found the same epithelial barrier degradation that occurred with allergen stimulation. After the knockdown or inhibition of IL-1R1, the effector receptor of IL-1*β*, we also observed considerable mitigation of the damage to the barrier when the nasal mucosal epithelial cells were stimulated again with allergens. Based on these results and combined with the upstream and downstream signaling pathways of IL-1*β* mentioned in previous studies, we tentatively summarize its mechanism in AR, as shown in Figure 2. Certainly, there may be other mechanisms, which need to be further investigated. To sum up, IL-1*β* plays a critical role in the development, maintenance, and progression of AR. Blocking the IL-1*β* pathway may be a new strategy for suppressing or controlling the occurrence and development of AR, and clinical study on IL-1*β*-targeted drugs in AR patients is urgently needed.

In conclusion, IL-1*β* is closely related to the occurrence and development of AR, with shared and unique roles when compared with other allergic diseases. In AR, IL-1*β* affects the body through different signaling pathways and generates corresponding immune responses. Many studies have explored the role and mechanism of IL-1*β* in cell and animal models of AR. The IL-1*β*-blocking treatment has been attempted; in human subjects, however, only the changes in IL-1*β* expression have been observed, but no clinical interventional trial or in-depth study on the mechanism has been carried out explicitly. On the one hand, the translation of basic research to clinical diagnosis and treatment of AR requires more prospective cohort studies; on the other hand, further research on molecular mechanisms should be conducted to promote early clinical translation and application in this field.

## Figures and Tables

**Table 1:** Expression of IL-1*β* in allergic diseases.

Disease	Allergen	Species of sample	Type of sample	IL-1*β* expression	Expression time to peak	References
Allergic rhinitis	Grass, birch, or hazelnut pollen	Human	Nasal secretions	Increases	5-6 h	Wagenmann et al., [11]
	Grass or ragweed	Human	Nasal mucosa	Increases	5 h	Sim et al., [12]
	Dust mite, dander, grass pollen, or ragweed pollen	Human	Nasal secretions	Increases	7 h	Weido et al., [13]
	Timothy grass pollen	Human	Nasal lavage	Increases	Unknown	Ivory et al., [15]
	Dactylis glomerata grass pollen	Human	Nasal lavages	Increases	2 h	Badorrek et al., [18]
	Grass pollen	Human	Nasal secretions	Increases	8 h	Bachert et al., [19]
	Ovalbumin	BALB/c mice	Serum and nasal mucosa tissues	Increase	Unknown	Kim et al., [39]
	Ovalbumin	Swiss albino mice	Serum	Increase	Unknown	Aswar et al., [40]
	House dust mite	Nasal epithelial cells	Cell extract	Increase	Unknown	Takizawa et al., [24]

Allergic asthmatics	Seasonal allergen	Human	Serum	Increase	Unknown	Karjalainen et al., [25]
	Inhalant allergens	Neonates	Airway mucosal lining fluid	Reduced	Unknown	Chawes et al., [26]
	Toluene diisocyanate	C57BL/6 mice	Lung tissue and blood	Increase	Unknown	Johnson et al., [46]
	Ovalbumin	BALB/c mice	Lung homogenates	Increase	Unknown	Jang et al., [41]
	House dust mite	C57BL6/NJ	Bronchoalveolar lavage and lung tissue	Increase	Unknown	Qian et al., [42]
	House dust mite	Human PBMCs and monocytic THP1 cells	Cell extract	Increase	Unknown	Wei et al., [43]

Atopic dermatitis	Ragweed	Human	Skin blisters	Increases	1 h, 11 h	Bochner et al., [44]
	Urushiol	Human	Epidermal tissue	Increases	6 h	Boehm et al., [14]
	House dust mite	Human	Epidermis	Increase	Unknown	Junghans et al., [27]
	Dinitrofluorobenzene	BALB/c mice	Epidermis	Increase	2 h	Enk et al., [17]
	Hexavalent chromium	HaCaT cells	Cell extract	Increase	Unknown	Wang et al., [45]

Allergic conjunctivitis	Seasonal allergen	Human	Tear	Increase	Unknown	Leonardi et al., [37]
	Ovalbumin	Rabbits	Corneas	Increase	Unknown	Zhao et al., [38]

Anaphylactic colonic	Cow's milk	Dunkin-Hartley Guinea pigs	Colon	Increase	Unknown	Theodorou et al., [21]

**Table 2:** Clinical test results and clinical applications of IL-1*β*.

Sample	Age (years)	Numbers (AR)	Sample source	Expression of IL-1*β* (pg/ml)		Clinical application	References
				Control	AR		
Children	6-10	292 (210)	Serum	0.13-0.69 (0. 23)	11.07-15.08 (13.42)	It is considered in the article as a significant predictor of the risk of moderate to severe AR	Han et al., [88]
	6-12	116 (116)	Blood	Unknown	12.45 ± 13.29	It is considered in the article as a significant predictor of the risk of moderate to severe AR	Han et al., [91]

Adults	21-29	12 (12)	Nasal secretions	Unknown	0.7-11.6 (8.0)	It is considered in the article that it can act as a signature factor for AR inflammation	Wagenmann et al., [11]
	Unknown	10 (10)	Nasal secretions	Unknown	19.5	It is used in the article as an indicator for assessing the efficacy of AR drugs	Weido et al., [13]
	20-49	20 (10)	Nasal lavage fluids	6.7 ± 1.9	8.5 ± 1.6	It is suggested in the article that it can be an index of the extent of neutrophil involvement in AR	Månsson et al., [57]
	Unknown	Unknown	Nasal secretions	0.2 ± 0.1	3.4 ± 2.6	It is used in the article as a distinguishing factor between AR and control	Bachert et al., [85]
	20-41	8 (4)	Serum	0.007 ± 0.014	0.583 ± 1.086	It is considered in the article that it can act as a signature factor for AR inflammation	Krouse et al., [90]
	15-66	41 (20)	Sera	0-412 (30)	3-996 (18)	It is used in the article as a distinguishing factor between AR and control	Gröger et al., [92]
	38 ± 16	137 (89)	Nasal fluid	4-1000 (20)	2-7894 (32)	It is considered in the article that it can act as a signature factor for AR inflammation	König et al., [93]
	29.42 ± 9.42	115 (85)	Plasma	Undetected-0.2 (0.13)	Undetected-1.72 (0.122)	It is used in the article as an indicator for assessing the efficacy of AR drugs	Bocşan et al., [94]

## Data Availability

Data sharing is not applicable to this article as no datasets were generated or analyzed during the current study.
